# Characterization of an Electronic Nicotine Delivery System (ENDS) Aerosol Generation Platform to Determine Exposure Risks

**DOI:** 10.3390/toxics11020099

**Published:** 2023-01-21

**Authors:** Qian Zhang, Jennifer Jeon, Travis Goldsmith, Marilyn Black, Roby Greenwald, Christa Wright

**Affiliations:** 1 Chemical Insights Research Institute, UL Research Institutes, Marietta, GA 30067, USA; 2 Department of Physiology and Pharmacology, West Virginia University/IEStechno, Morgantown, WV 26505, USA; 3 School of Public Health, Georgia State University, Atlanta, GA 30303, USA

**Keywords:** electronic cigarette, e-liquids, emissions, monitoring, particles

## Abstract

Evaluating vaping parameters that influence electronic nicotine delivery system (ENDS) emission profiles and potentially hazardous exposure levels is essential to protecting human health. We developed an automated multi-channel ENDS aerosol generation system (EAGS) for characterizing size-resolved particle emissions across pod- and mod-type devices using real-time monitoring instruments, an exposure chamber, and vaping parameters including different ventilation rates, device type and age, e-liquid formulation, and atomizer setup. Results show the ENDS device type, e-liquid flavoring, and nicotine content can affect particle emissions. In general, pod-type devices have unimodal particle size distributions and higher number emissions, while mod-type devices have bimodal size distributions and higher mass emissions. For pod-type devices, later puff fractions emit lower aerosols, which is potentially associated with the change of coil resistance and power during ageing. For a mod-type device, an atomizer with a lower resistance coil and higher power generates larger particle emissions than an atomizer with a greater resistance coil and lower power. The unventilated scenario produces higher particle emission factors, except for particle mass emission from pod-type devices. The data provided herein indicate the EAGS can produce realistic and reproducible puff profiles of pod- and mod-type ENDS devices and therefore is a suitable platform for characterizing ENDS-associated exposure risks.

## 1. Introduction

Electronic nicotine delivery systems (ENDS) have obtained rapid acceptance and a rise in popularity since 2006 in the United States consumer market [1] and are claimed to be safer than tobacco cigarettes and used as smoking cessation aids. However, there are no federal guidelines to ensure the relative safety of ENDS device usage behavior that may influence vapor formation, concentration, and composition [2,3,4], nor to ensure manufacturers provide adequate user information regarding maintenance or care of ENDS devices or e-liquids as they are used. This could contribute to unintentional exposure to hazardous byproducts [5]. ENDS consist of four fundamental components, including a mouthpiece, a fluid reservoir (either a cartridge or tank), an atomizer (a wick and a heating element called a vaporizer), and an energy source (battery) [6]. ENDS deliver nicotine by heating and vaporizing an e-liquid, which typically contains propylene glycol (PG), vegetable glycerin (VG), nicotine, and flavorings [7].

One of the most popular types of ENDS devices is called the pod-type device, which has a growing market share and is especially popular among adolescents [6,8] The pod-type ENDS devices possess and provide a simple device design, ease of use, and higher accessibility compared to other types of ENDS devices [9]. Moreover, the use of benzoic acid as a protonating agent in the pod-type e-liquids, e.g., JUUL pods, can provide higher nicotine delivery with less respiratory irritation [10]. Previously, several mod-type (i.e., a device with a refillable tank) ENDS studies showed that user-modifiable features could increase health risks from hazardous chemical compounds because of varying power and device configurations [11,12,13,14,15]. On the contrary, the pod-type ENDS devices include automatic temperature regulation features and a closed system, which restrict users from modifying the setups [6,16,17] However, a substantial amount of PM and VOCs are still detected from the pod-type ENDS-emitted aerosol [7], which could negatively affect users’ health [18]. Moreover, the level of exposure to these hazardous chemical compounds varies with several influential factors, including the device usage behavior of ENDS users [3].

Studies have found that ENDS emit high levels of particulate matter (PM), especially PM_2.5_ (smaller than 2.5 µm in size), and volatile organic compounds (VOCs). Reported ENDS aerosol emissions were primarily smaller than 1 µm [19,20,21,22], which also depended on e-liquid composition [23,24] and vaping conditions like puff flow rate and puff number, as well as environmental conditions [7,19,20,22,25,26] With the increase in use of ENDS, especially among youth globally [27], adverse respiratory health outcomes in users have been reported [28]. In addition to potential toxicity and adverse health effects related to mainstream and secondhand ENDS vaping, ENDS use has become a concern for public health [7,29,30,31]. Importantly, recent studies have revealed that 70% of inhaled ENDS aerosols generated during mainstream vaping are subsequently exhaled, which contributes to secondhand exposure [32]. Mainstream vaping may also contribute to secondhand ENDS aerosol or emission exposures because of the perception that ENDS are safer to use in indoor environments than traditional tobacco products. While secondhand vaping aerosols contain fewer hazardous compounds than tobacco-based secondhand smoking aerosols, bystanders in close proximity to vaping individuals may be exposed to harmful constituents including ultrafine particles, VOCs, and heavy metals [33,34].

Particle size distribution and concentration are essential factors in estimating the potential adverse health effects of vaping [35]. However, these variables are highly influenced by device usage behavior [3,36,37], and monitoring conditions like air change rate, temperature, and relative humidity [3,7]. Therefore, the characterizations of ENDS emissions tend to be dependent on study design, which could lead to uncomparable or inconsistent results among studies [19,38,39]. The capability to generate ENDS aerosol in a controlled manner is critical in characterizing ENDS aerosol properties and to determine differential exposure [40,41]. In this paper, we developed a comprehensive methodology for characterizing ENDS aerosol emissions using a custom-made automated ENDS aerosol generation system (EAGS) that can be applied with various ENDS device types and customized settings. We used an exposure chamber setup and online particle measuring instruments to monitor emitted particle concentration and size distribution for ultrafine, fine, and coarse sizes, under simulated ventilated and unventilated environments. In addition, the method was applied to investigate the influences of different parameters on calculated particle emission factors, which included ENDS device type, e-liquid flavoring, atomizer setting, and puff fraction (atomizer ageing).

## 2. Methods and Materials 

### 2.1. ENDS Device and E-Liquid Selection

Two types of commonly used ENDS devices, pod-type and mod-type, were studied. The devices were chosen based on their web-based reviews, popularity, and availability in local stores. The pod-type device, JUUL, contains a pre-filled cartridge with specific favored e-liquid, while the mod-type device, VOOPOO DRAG 2, contains a refillable tank and replaceable atomizer that allows users to change atomizer setups and e-liquid flavors on the same device. The setup parameters of the studied ENDS devices, as well as the e-liquid flavors, are listed in Table 1; power is the maximum power of the device measured by EAGS. For each type of device, there were two different e-liquid flavors studied. Tobacco 1 (Virginia Tobacco) and 2 (Classic Tobacco) that were used with JUUL pods were from JUUL with a PG/VG ratio of 30/70. Tobacco 3 (American Patriots) and 4 (Rothschild Apple Tobacco) were two brands applied with mod-type devices, with a PG/VG ratio of 35/65 for Tobacco 3 and an unknown ratio for Tobacco 4. 

### 2.2. ENDS Aerosol Generation System

**Figure 1:**
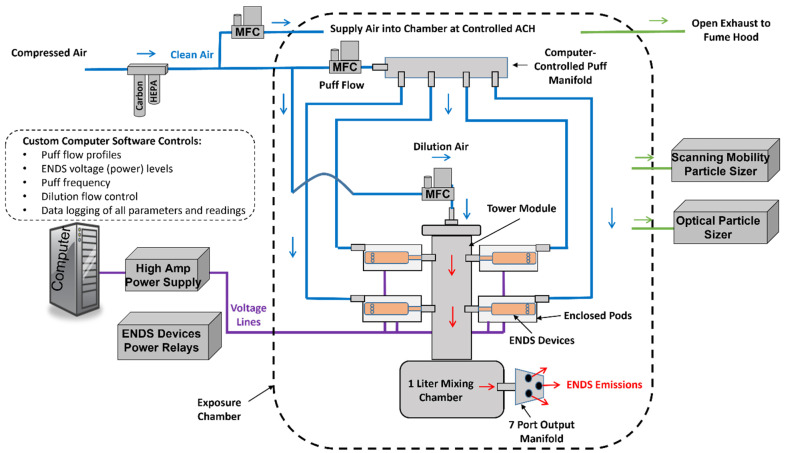
Fully automated multi-channel ENDS aerosol generation system, set up in an exposure chamber with aerosol characterization instruments. Pressurized air is cleaned and passed through “puff” and dilution mass flow controllers. Pods in the tower module each house an individual ENDS device. The puff signal is fed from the manifold into the specific pod while a voltage is also applied to the pod to activate the ENDS device. The puff then passes into the tower module where it is mixed with the dilution air then further mixed in a stainless-steel mixing chamber. The output emissions leave the generator through a multi-port manifold where they are either sampled directly or released into the exposure chamber where they are diluted with clean air and sampled over an extended period of time.

A custom-built four-channel automatic ENDS aerosol generation system (EAGS) (IEStechno, WV, USA) was designed and constructed to generate ENDS emissions under controlled conditions. A diagram of the system is shown in Figure 1. Clean pressurized air was supplied to two high-speed mass flow controllers (MFC) (Alicat, MC). The “puff” MFC provided a user-defined flow signal that simulated a single human puff. This flow was fed into a four-channel manifold that consequently supplied the flow signal into one of four enclosed polycarbonate pods that were coupled into a tower module. Each pod encapsulated a separate ENDS device. The flow signal was pushed through the ENDS device and out the mouthpiece that was coupled to the tower. A high amperage (40 A) computer-controlled DC power supply (Acopian, Y08LXB4000) supplied the voltage to the particular ENDS device through a power relay bank attached to 510 connectors in each pod (the batteries were removed). The power supply also measured the real-time voltage and current that was supplied to each ENDS device, which allowed the wattage to be calculated for each puff. The voltage was applied at the same time that the flow signal was passed through each individual device, which resulted in ENDS emissions into the tower module. Dilution air was passed through the “dilution” MFC and into the top of the module where it mixed with the ENDS emissions. A conceptual lung, a one-liter stainless steel mixing chamber, was situated at the bottom of the module to simulate aerosol mixing and deposition in the lungs. The mixed emissions then passed through a custom stainless steel output manifold with seven ports into the various sampling instruments and/or exposure chamber. Up to four of the pods with ENDS devices could be used during each experiment. Each device was activated independently of the others and the emissions from all were added to the tower module. This allowed high output (emissions from up to four devices) with an acceptable amount of time for each individual device to cool down before reactivating. 

Various types of ENDS devices can be utilized in the EAGS with the 510 connectors and adaptors to generate aerosols with user-defined voltage/wattage delivery. The EAGS was controlled by the IEStechno software, which allowed adjustable voltages (0–8 V), puff flows (0–5 LPM), dilution air flows (0–20 LPM), and custom puff profiles. The puff profile for each ENDS device in this study consisted of a square wave with a 55 mL volume and 3 s duration, following the Center for Scientific Research Relative to Tobacco (CORESTA) Recommended Method No.81 [41]. Dilution flows of 1.25 to 5 LPM with a puff flow of 1.1 LPM (based on the CORESTA profile) and puff rates of 0.2–4 puff/min were applied in this study.

### 2.3. Exposure Chamber Environment

The generated emissions were released into a 6 m^3^ stainless steel exposure chamber to provide additional dilution before aerosol measurements, which also served as a conditioning environment for secondhand vaping exposure evaluation. The exposure chamber was designed and validated for its airtightness, mixing and chemical recovery according to standards [42,43]. An MFC (Aalborg, GFC67) controlled the clean air supply into the chamber at a set air exchange rate (ACH). Sampling ports for particle characterizations were located on chamber walls and connected to the instruments outside of the chamber (Figure 1). 

To assess the ventilation effect, we characterized particle emissions under two ventilation scenarios using the exposure chamber. One is an unventilated room scenario. The exposure chamber was set up with a 0 ACH and a mixing fan inside; EAGS dilution flow was adjusted to compensate for instrument sampling flow. This static chamber setup was used to characterize ENDS emissions from small puff numbers. Tobacco 1 (5% nicotine strength) with the pod-type device (coil resistance = 2.0 Ω, power = 7 W) and Tobacco 3 (0.3%) with the mod-type device (coil resistance = 0.2 Ω, power = 45 W) were characterized under the static chamber setup with puff numbers of 20 (pod) and 4 (mod) depending on OPS measurement capability. Extended puff intervals (up to 80 min) were applied for the static chamber setup to enable evaluation of emissions from each puff. 

The other scenario is a ventilated environment with a filtered air supply. The dynamic chamber ran at 3 h^−1^ ACH and was applied to characterize emissions from large puff numbers (over 100 puffs) with higher puff rates. Additional samples were collected from the EAGS output manifold for toxicological analysis described in a companion paper [44] and the rest of the emission was released inside the exposure chamber. All studied ENDS devices and e-liquids were characterized with the dynamic chamber setup with various puff numbers to evaluate the influences of puff conditions. Detailed setups of each experiment are listed in Supplementary Materials Tables S1 and S2. 

### 2.4. Real Time Particle Monitoring and Emission Characterization

Particle concentration and size distribution inside the exposure chamber were measured with a scanning mobility particle sizer (SMPS, models TSI 3080 and 3786) for particles between 7 to 300 nm size and an optical particle sizer (OPS, TSI 3330) for 0.3–10 µm size particles. Total particle emission (*TP*) was calculated using Equation (1) based on a previously developed emission testing method [45,46].

(1) $$TP=\sum_{t_{start}}^{t_{stop}}\left(V_{c}\left(\frac{C\left(t\right)-C\left(t-∆t\right)e^{-\beta ∆∆t}}{∆t\times e^{-\beta ∆∆t}}\right)\times ∆t\right)$$

where *t_start_* and *t_stop_* indicate the time duration of emission; *V_c_* is the volume of the chamber; *C* is particle concentration at a given time *t*; Δ*t* is the time interval of particle sampling; *β* is the loss coefficient calculated from particle decay after vaping stopped. Furthermore, emission factor (*EF*) was calculated using Equation (2).

(2) $$EF=\frac{TP}{puff\;number}$$

For a dynamic chamber setup with additional sampling, a dilution factor (dilution factor = flow rate into EAGS/ flow rate into exposure chamber) was applied to convert chamber measurements into EAGS emissions. Particle emission per puff (i.e., emission factor) was calculated for both particle number (#) and mass emissions, assuming spherical particles with density of 1 g/cm^3^.

### 2.5. Statistical Analysis

The t-test was used to compare the results of two different groups with a *p*-value of 0.05. The number of replicates is provided within each figure, and all calculations were performed in GraphPad Prism (9.3.1).

## 3. Results

### 3.1. Particle Emissions from Different Devices under Two Ventilation Scenarios 

**Figure 2:**
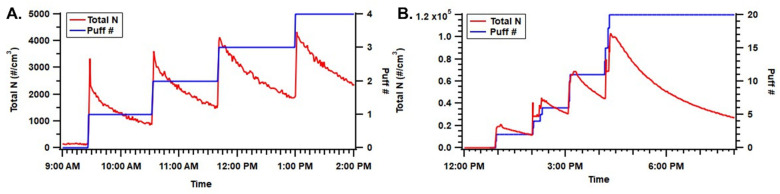
Particle number concentration inside the static chamber and puff number as a function of time. Panel (**A**) presents the mod-type device with four puffs and panel (**B**) presents the pod-type device with 20 puffs.

In the static chamber (unventilated) condition, particle concentrations increased corresponding to the number of puffs generated (Figure 2). For the mod-type device, the total number of puffs evaluated was four, and they were evaluated once per hour to avoid OPS measurement exceeding the maximum detection limit (3000 #/cm^3^). For the pod-type device, the total puff number evaluated was 20, and they were grouped into four groups at one hour sampling per group. The mod-type device generated lower particle-number concentrations, which were associated with a smaller puff number. In addition, the mod-type device had a relatively larger particle-loss coefficient (2.20 × 10^−4^ #/s on average) compared to the pod-type device (1.34 × 10^−4^ #/s on average), which resulted in a quicker decay of particle number concentration and less accumulation of particles inside the chamber (Figure 2). 

**Figure 3:**
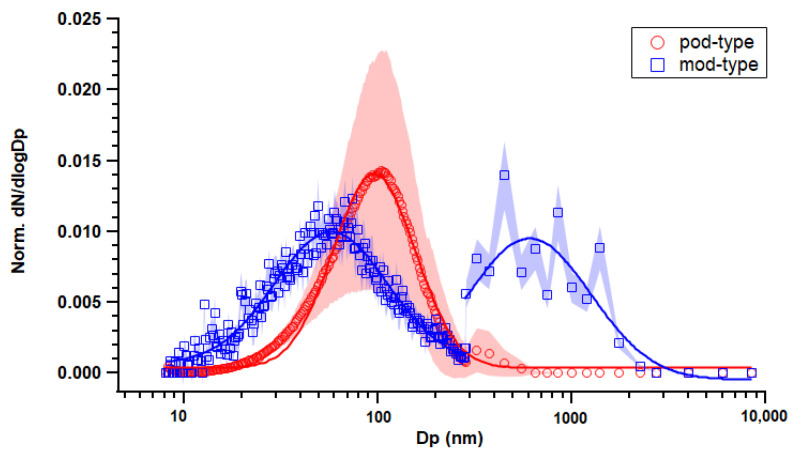
Normalized particle number size distribution of aerosol emissions from pod-type and mod-type devices in the static chamber. Marker indicates averaged data throughout the puff duration (4 puffs for mod-type and 20 puffs for pod-type); shade indicates standard error; line indicates fitted lognormal curve.

The pod-type device created a particle size distribution with one mode at approximately 100 nm (Figure 3). Larger deviations were observed that were due to the accumulation of small particles through an increase in puff numbers; in addition, the geometric mean diameter (GMD) of the size distribution shifted slightly to a larger size, from 67.9 nm to 96.8 nm on average, which could be due to vapor condensation and particle–particle coagulation. On the other hand, for mod-type device emissions, there were two separate modes in particle number distribution (Figure 3): one in Aitken mode (58.2 nm) and another one in accumulation mode (794 nm). Bi-modal aerosol size distributions have also been reported previously for mod-type devices [21,24,34]. The larger-sized particles could contribute to the larger loss coefficient caused by particle deposition. 

The calculated emission factors showed a larger number of emissions from pod-type devices but larger-mass emissions from mod-type devices (Table 2). This is associated with the different size distributions from each device, where smaller particles contributed largely to the number of emissions and larger particles contributed more to the mass of emissions. Melstrom et al. [35] also found mod-type devices had higher PM_2.5_ emissions with lower ultrafine particle emissions than pod-type devices. The identical vaping setup for each device type was also run under the ventilated (dynamic chamber) scenario to evaluate the influence of experimental condition on particle emission characterizations from ENDS. Instead of directly comparing particle concentration levels inside the chambers that were affected by dilution, the calculated emission factors that accounted for particle loss and dilution factor are listed in Table 2 for comparison. The unventilated scenario had three to nine times higher particle-number EFs for mod- and pod-type devices, respectively. For particle-mass EF, the unventilated scenario was 14 times higher for mod-type devices while the ventilated scenario was 7 times higher for pod-type devices. This indicated the effect of dilution on aerosol dynamics could contribute to the variation of particle emission characterization. The average particle mass emission was 0.26–1.71 mg/puff from pod-type devices and 1.09–15.0 mg/puff from the mod-type devices, which is comparable to the range reported by Gillman et al. (1.5–28 mg/puff) [47].

### 3.2. Differences in E-Liquid Formulation Modulate Particle Emissions

**Figure 4:**
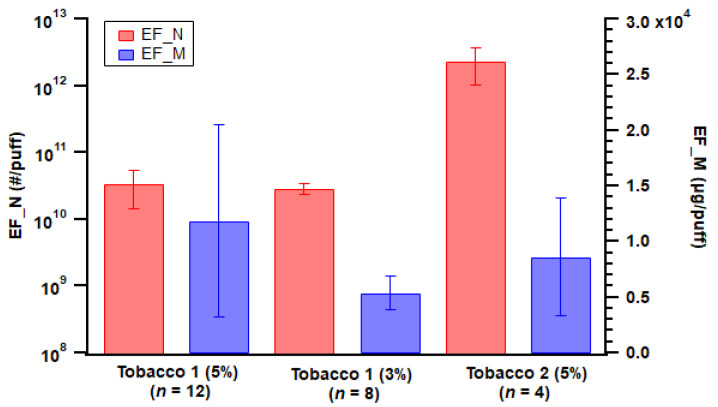
Particle emission factors of both number (EF_N) and mass (EF_M) from the three different e-liquid flavors used with the pod-type device. Error bar indicates standard error; *n* = number of replicates.

E-liquid flavor could potentially affect ENDS emissions. For pod-type devices, Tobacco 2 flavor emitted two orders of magnitude more particle numbers than the two Tobacco 1 flavors (Figure 4), which could be associated with semi-volatile compounds derived from flavoring additives forming new particles. However, Manigrasso et al. reported no effects of flavors on particle concentrations [48]. Tobacco 1 with 3% of nicotine strength had a lower average particle mass emission, while overall the mass emissions from the three flavors were comparable (Figure 4). In addition, Tobacco 2 flavor generated aerosols with a GMD of 113 nm on average, while for Tobacco 1 flavor it was 359 nm (5% nicotine strength) and 247 nm (3%) on average. This indicated Tobacco 2 flavor e-liquid emitted smaller particles in general while not necessarily contributing largely to mass emissions. 

**Figure 5:**
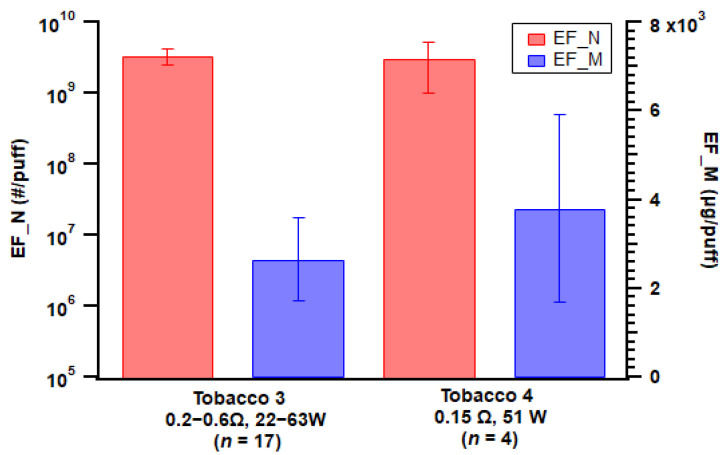
Particle emission factors of both number (EF_N) and mass (EF_M) from the two different e-liquid flavors applied with the mod-type device. Error bar indicates standard error; *n* = number of replicates.

For the mod-type device, two tobacco flavors with the same nicotine strength generated comparable levels of particle number and mass emissions (Figure 5), which is consistent with Manigrasso et al. [48]. However, it should be noted that the atomizer setup varied for devices with different e-liquids (see Table 1).

### 3.3. Device Ageing and Puff Fraction Number alter ENDS Particle Profiles

**Figure 6:**
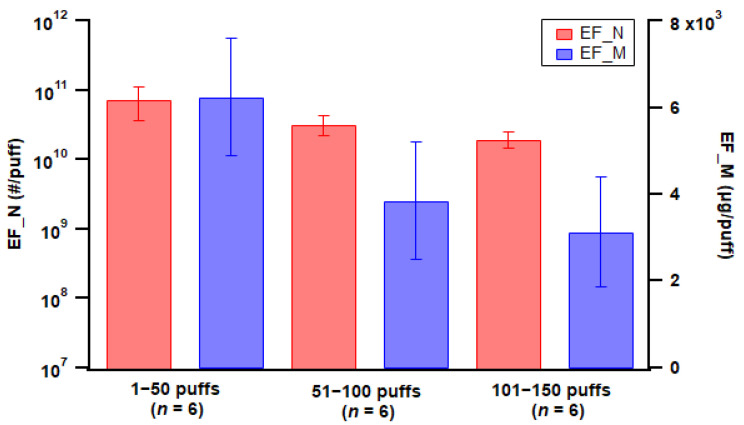
Particle emission factors of both number (EF_N) and mass (EF_M) for the three puff fractions using the pod-type device with Tobacco 1 flavor e-liquid. Error bar indicates standard error; *n* = number of replicates.

Puff fraction was classified into initial (1–50 puffs), middle (51–100 puffs), and late (101–150 puffs) fractions, considering the pod-type device studied was nearly depleted after 150 puffs. Emission factor calculations showed a slight decrease in particle number emissions with increased puff fraction (Figure 6), which could be associated with particle coagulation reducing particle numbers inside the chamber; however, the differences among puff fractions were not statistically significant. Similarly, particle mass EF also decreased with later puff fractions (Figure 6), while the differences were not statistically significant. However, for mod-type devices, there was no consistent trend for device ageing; measurement showed an increase in particle emissions with ageing for some cases while showing a decrease for other cases (see Figures S1–S5). 

### 3.4. Influence of Atomizer Format on Particle Emissions

**Figure 7:**
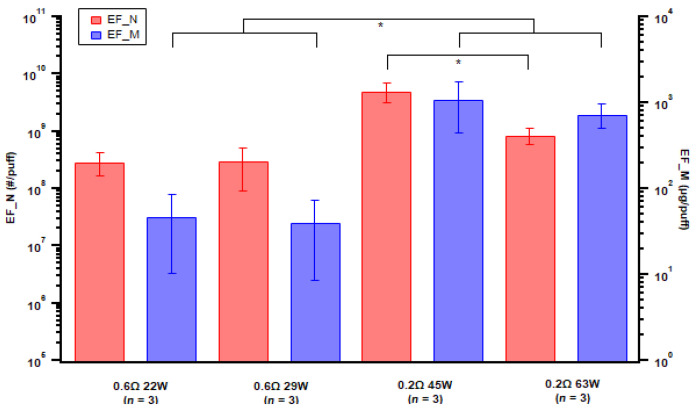
Particle emission factors of both number (EF_N) and mass (EF_M) for various atomizer setups. Error bar indicates standard error; *n* = number of replicates. Asterisk indicates *p*-value < 0.05.

For atomizer format, we examined two coils with different resistance and for each coil with two different power setups using the same e-liquid (Table 1). The emission factors were comparable when an 0.6 Ω coil was used with different wattage, but an increase in number EF was noted from an 0.2 Ω coil with 45 W power (Figure 7). The lower resistance coil generated higher particle-mass emissions than the higher resistance coil (Figure 7), which was also associated with higher power in general; the difference between the mass emission factors from the two coils was statistically significant (*p*-value = 0.047). 

## 4. Discussion and Limitations

Particle emissions from ENDS devices are affected by a combination of various parameters including e-liquid preference and device format and age. Alongside device parameters, evaluating mediators of exposure such as ventilation in defined spaces is also necessary to determine the human health impacts of ENDS exposures. Secondhand vaping exposures to ENDS aerosols are an understudied but important component in determining the relative safety of products that are touted as safer than tobacco cigarettes. Due to this common misconception on ENDS safety, vaping in poorly ventilated spaces could occur more frequently and contribute to and/or exacerbate respiratory conditions in children and adults. Indeed, recent evidence suggests secondhand vape exposure is associated with an increased risk of bronchitis and shortness of breath in young adults [49]. Furthermore, in 2019, 6.7 million American middle and high school students reported exposure to secondhand vape or smoke [50]. Thus, when combined with other indoor air quality issues (i.e., mold, dust, viruses, etc.), ENDS usage and secondhand vaping exposures may create potential unknown respiratory health concerns. 

Previous studies have shown that ENDS aerosol emissions were associated with e-liquid compositions, especially PG/VG ratio [51]. Higher particle-mass emissions and fewer numbers of emissions were observed with an increase in PG/VG [23], which is also observed in this study (Table 2). However, the relationship between PG/VG ratio and aerosol emissions tended to also depend on environmental conditions during sampling, including air exchange rate, temperature, relative humidity, and dilution factor [52]. For example, Li et al. observed a decrease in PM_2.5_ emission with higher PG/VG that was due to air exchange rates within the testing environment [52]. In addition, nicotine content and form could also affect the evaporation of e-liquid and emitted aerosols. We found that the pod-type device with 5% protonated nicotine e-liquid emitted more particles in number compared to the mod-type device. This could be related to the fact that protonated nicotine vaporizes more readily than previous formulations, which produces secondary aerosols because of vapor condensation. However, Talih et al. found nicotine form does not affect emission of total particle mass [53]. The size of ENDS-emitted aerosols is influenced by the evaporation/condensation of water vapor, volatile and semi-volatile compounds generated from e-liquids during vaping, which is sensitive to the dynamics of air conditions. Specifically, PG evaporates quicker than VG [51,52], thus a higher PG/VG ratio could lead to aerosols shrinking to smaller sizes [23]. The aerosols emitted from the mod-type device had a smaller GMD than that from the pod-type device (Figure 3), which could be associated with its higher PG/VG ratio in the e-liquid. However, another mode in a larger size was also observed for the mod-type device, which could be attributed to the water uptake of aerosols when they grow larger than the critical size, given both PG and VG are hygroscopic [38]. The effect of nicotine strength on aerosol emissions was not conclusive; studies have reported nicotine associated with an increase in PM emissions [19,48] or a decrease in particle number and mass emissions [24,52] for mod-type devices. In addition, nicotine concentration tended not to affect the size of emitted aerosols [24,48].

**Figure 8:**
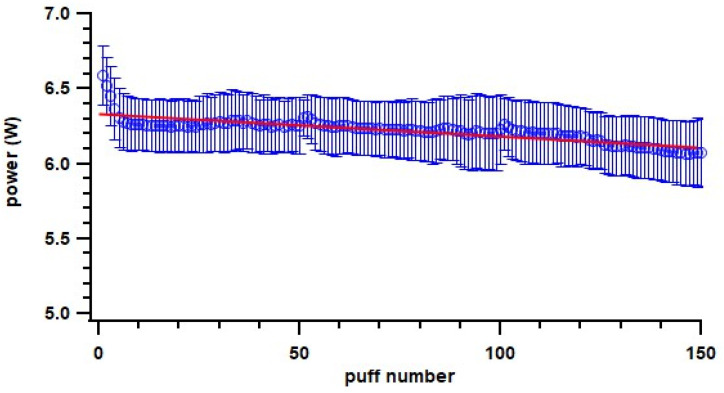
Change of power on pod-type ENDS devices with increase in puff number. Blue circle indicates measured power; error bar indicates standard deviation; line indicates fitted linear curve.

Various studies have showed particle mass emissions that increased with higher power because of larger PG/VG mass consumption [25,47,54,55], which is consistent with the overall trend in this study that a 0.2 Ω coil emitted higher particles than a 0.6 Ω coil. However, for the same coil resistance, an increase in power seemed to have no effect or a decreasing trend on particle emissions. Floyd et al. observed complex influences of power on particle emission; the increase in power was associated with an increase in particles larger than 600 nm but a decrease in particles smaller than 600 nm [25]. In addition, there were potentially other factors influencing measured particle emissions, with air dynamics (e.g., dilution) likely affecting condensation and evaporation processes [52]. This was also indicated when comparing the static chamber (unventilated environment) to the dynamic chamber (ventilated environment) setup. The particle number and mass EFs from the static chamber were higher than from the dynamic chamber for the same ENDS device and e-liquid setup, except the mass EF of the pod-type device. For the pod-type devices, we found a decrease in particle emissions with later puff fractions; one possible reason could be the power applied on the coil decreased through the puff fractions (Figure 8) with the consumption of e-liquid. Specifically, the correlation coefficient of particle mass EF with power is 0.73. The pod-type device is designed to achieve consistencies of vaping conditions; one potential reason for power decreasing with device ageing could be the heating coil resistance increasing with ageing (power = voltage^2^/resistance if voltage is constant), which could be a result of the coil shedding material during use and thus becoming thinner. Another potential reason could be associated with the coil and e-liquid temperature. Power seemed to be the highest at the beginning of each puff fraction when the coil and e-liquid were cool (Figure 8) and then decreased when they became warmer, which likely increased overall resistance. 

One limitation of this study is that the dilution factor varied for different vaping experiments to compensate for additional sampling for a different study; therefore, the effect of the dilution factor on aerosol dynamics and further overall emissions was not conclusive. We found that the experimental condition, e.g., dilution, had an impact on measured and calculated emission factors for the same vaping condition. Therefore, it is difficult to compare emission results reported from different studies even for the same vaping device and e-liquid used, or factors influencing emissions; a standardized emission characterization protocol may be needed. Additionally, while our findings suggest ventilation may reduce ENDS particle levels, the health hazards associated with vaping are still under debate; therefore, vape-free or smoke-free environments are recommended to protect public health. 

## 5. Conclusions

In this study, a custom-built fully automated multi-channel ENDS aerosol generation system was utilized to demonstrate real-life vaping scenarios by applying pertinent parameters in a systemic and controlled environment. This platform allows characterizations of aerosol emissions from various ENDS device types, which can be used to assess toxicological properties of emitted aerosols and further link emission properties to potential health impacts. We evaluated the EAGS platform by applying two types of ENDS devices with different flavored e-liquids and atomizer setups, measuring particle concentration and size distribution for different puff fractions. We found that particle emission factors were generally higher in mass for the mod-type device and higher in number for the pod-type device. The observed particles emitted were typically in ultrafine and fine sizes, with the pod-type device generating single-modal while the mod-type device generating bi-modal distributions. Vaping conditions like e-liquid flavor and nicotine strength, atomizer power and coil resistance, and puff fraction potentially had influence on measured particle emissions. In addition, sampling conditions such as air flow and dilution also influenced aerosol emissions.

## Figures and Tables

**Table 1:** Evaluated ENDS devices and e-liquids.

ENDS Device	E-Liquid Flavor (Nicotine Strength)	Coil Resistance (Ω)	Power (W)
Pod-type	Tobacco 1 (5%)	2.0	7
	Tobacco 1 (3%)	2.0	7
	Tobacco 2 (5%)	2.0	7
Mod-type	Tobacco 3 (0.3%)	0.2	45
	Tobacco 3 (0.3%)	0.2	63
	Tobacco 3 (0.3%)	0.6	22
	Tobacco 3 (0.3%)	0.6	29
	Tobacco 4 (0.3%)	0.15	51

**Table 2:** Particle emission factors (average ± standard error) for pod-type and mod-type devices for two ventilation scenarios.

	Pod-Type	Mod-Type
Unventilated scenario (*n* = 4)		
Number emission (#/puff)	4.92 × 10^10^ ± 0.70 × 10^10^	1.72 × 10^10^ ± 0.08 × 10^10^
Mass emission (µg/puff)	2.58 × 10^2^ ± 0.60 × 10^2^	1.50 × 10^4^ ± 0.06 × 10^4^
Ventilated scenario (*n* = 3)		
Number emission (#/puff)	5.54 × 10^9^ ± 2.11 × 10^9^	5.00 × 10^9^ ± 1.91 × 10^9^
Mass emission (µg/puff)	1.71 × 10^3^ ± 1.00 × 10^3^	1.09 × 10^3^ ± 0.64 × 10^3^

## Data Availability

The data presented in this study are available upon request from the corresponding author.

## Supplementary Materials

The following supporting information can be downloaded at https://www.mdpi.com/article/10.3390/toxics11020099/s1: Table S1. Puff conditions for pod-type devices under dynamic chamber setup; Table S2. Puff conditions for mod-type devices under dynamic chamber setup. Figures S1–S5. Particle emission factors of number (EF_N) and mass (EF_M) for different puff fractions with various mod-type device setups.
