# Supplementary material for: Characterization of an Electronic Nicotine Delivery System (ENDS) Aerosol Generation Platform to Determine Exposure Risks
Source: Toxics. 2023 Jan 21;11(2):99. doi: 10.3390/toxics11020099 (PMC9967066; doi:10.3390/toxics11020099)
Supplement: Supplementary file 1 [file toxics-11-00099-s001.zip › toxics-2132788-supplementary.pdf]

Supplemental Materials

Table S1. Puff conditions for pod-type devices under dynamic chamber setup.

#	# of pod	e-liquid	dilution factor	puff number for each pod	puff rate (#/min)
1	2	Tobacco 1 (5%)	1.11	1-50	2
	2	Tobacco 1 (5%)	1.11	51-100	2
	2	Tobacco 1 (5%)	1.11	101-150	2
	2	Tobacco 1 (5%)	1.11	151-200	2
2	3	Tobacco 2 (5%)	2.94	1-24	2
	3	Tobacco 2 (5%)	1.67	40-100	2
	3	Tobacco 2 (5%)	1.67	101-150	2
	3	Tobacco 2 (5%)	1.67	151-200	2
3	2	Tobacco 1 (3%)	1.67	1-50	2
	2	Tobacco 1 (3%)	1.67	51-100	2
	2	Tobacco 1 (3%)	1.67	101-150	2
	2	Tobacco 1 (3%)	1.67	151-200	2
4	1	Tobacco 1 (3%)	5.00	1-60	2
	1	Tobacco 1 (5%)	5.00	1-120	4
5	3	Tobacco 1 (5%)	7.67	1-50	2
	3	Tobacco 1 (5%)	7.67	51-100	2
	3	Tobacco 1 (5%)	7.67	101-150	2
6	3	Tobacco 1 (5%)	7.67	1-8	2
7	3	Tobacco 1 (5%)	21	1-50	2
	3	Tobacco 1 (5%)	1	51-100	2
	3	Tobacco 1 (5%)	21	101-150	2

8	1	Tobacco 1 (5%)	1	1-62	0.5
9	1	Tobacco 1 (5%)	21	1-50	2
	1	Tobacco 1 (5%)	1	51-100	2
	1	Tobacco 1 (5%)	21	101-150	2
10	1	Tobacco 1 (3%)	21	1-50	2
	1	Tobacco 1 (3%)	1	51-100	2
	1	Tobacco 1 (3%)	21	101-150	2

Table S2. Puff conditions for mod-type devices under dynamic chamber setup.

#	e-liquid	coil resistance (Ω)	power (W)	dilution factor	puff number	puff rate (#/min)
1	Tobacco 3 (0.3%)	0.6	22	1.91	1-50	2
	Tobacco 3 (0.3%)	0.6	22	1	51-100	2
	Tobacco 3 (0.3%)	0.6	22	1	101-150	2
	Tobacco 3 (0.3%)	0.6	22	1	151-200	2
2	Tobacco 4 (0.3%)	0.15	51	1.91	1-50	2
	Tobacco 4 (0.3%)	0.15	51	1	51-100	2
	Tobacco 4 (0.3%)	0.15	51	1	101-150	2
	Tobacco 4 (0.3%)	0.15	51	1	151-200	2
3	Tobacco 3 (0.3%)	0.15	51	1.91	201-250	2
4	Tobacco 3 (0.3%)	0.2	45	21	1-25	0.3
	Tobacco 3 (0.3%)	0.2	45	21	101-125	0.3
	Tobacco 3 (0.3%)	0.2	45	21	201-225	0.3
5	Tobacco 3 (0.3%)	0.6	22	21	1-25	0.3
	Tobacco 3 (0.3%)	0.6	22	21	101-125	0.3
	Tobacco 3 (0.3%)	0.6	22	21	201-225	0.3

6	Tobacco 3 (0.3%)	0.2	63	3	1-25	0.3
	Tobacco 3 (0.3%)	0.2	63	3	101-125	0.3
	Tobacco 3 (0.3%)	0.2	63	3	201-225	0.3
7	Tobacco 3 (0.3%)	0.6	29	3	1-25	0.3
	Tobacco 3 (0.3%)	0.6	29	3	101-125	0.3
	Tobacco 3 (0.3%)	0.6	29	3	201-225	0.3

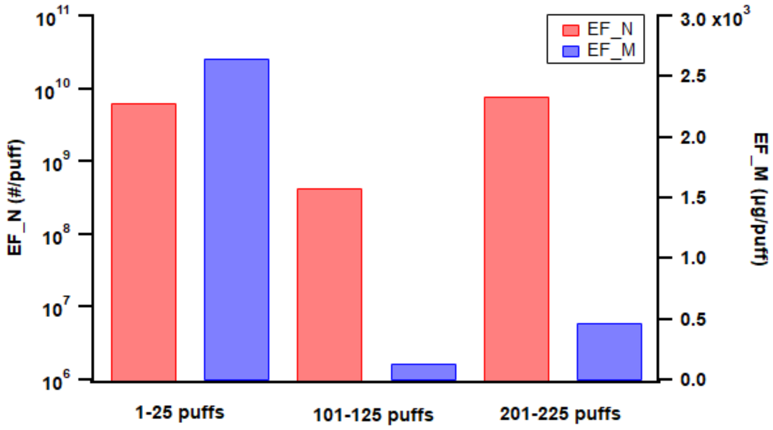

Figure S1. Particle emission factors of number (EF_N) and mass (EF_M) for the different puff fractions using the mod-type device (0.2 Ω, 45 W) with Tobacco 3 flavor e-liquid. Data from one experiment.

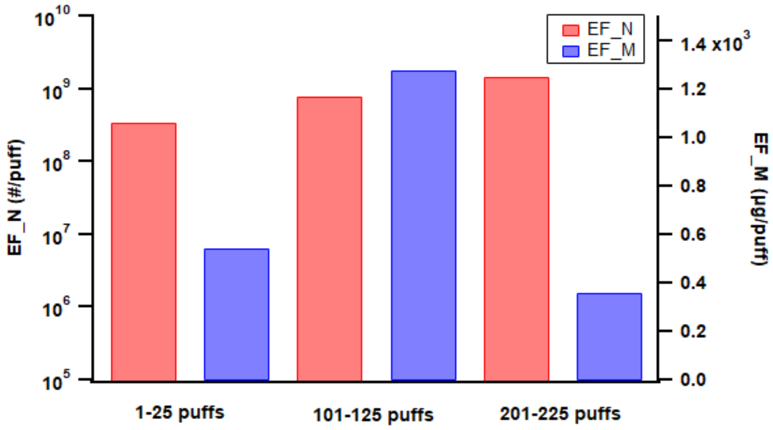

Figure S2. Particle emission factors of number (EF_N) and mass (EF_M) for the different puff fractions using the mod-type device (0.2 Ω, 63 W) with Tobacco 3 flavor e-liquid. Data from one experiment.

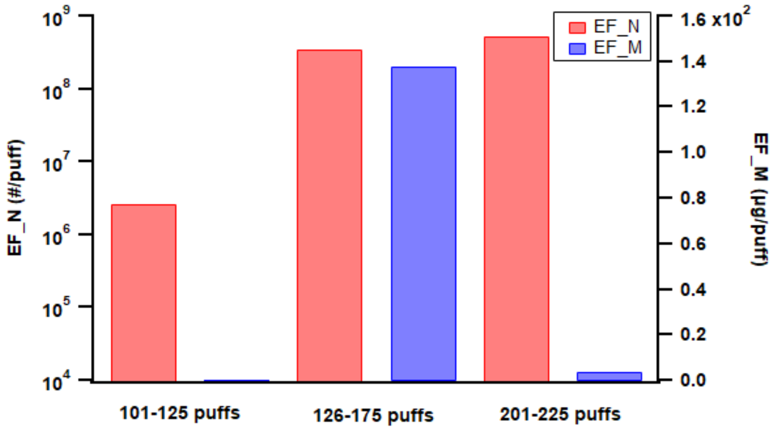

Figure S3. Particle emission factors of number (EF_N) and mass (EF_M) for the different puff fractions using the mod-type device (0.6 Ω, 22 W) with Tobacco 3 flavor e-liquid. Data from one experiment.

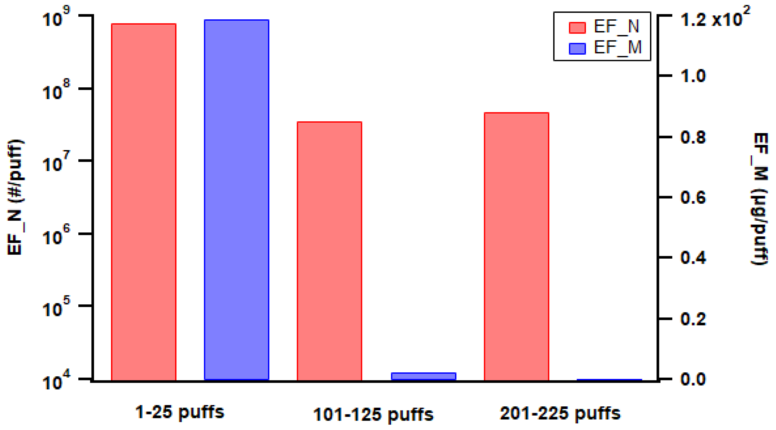

Figure S4. Particle emission factors of number (EF_N) and mass (EF_M) for the different puff fractions using the mod-type device (0.6 Ω, 29 W) with Tobacco 3 flavor e-liquid. Data from one experiment.

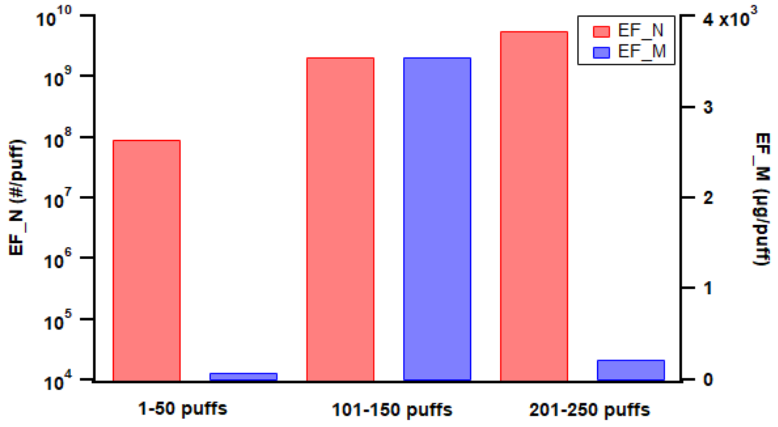

Figure S5. Particle emission factors of number (EF_N) and mass (EF_M) for the different puff fractions using the mod-type device (0.15 Ω, 51 W) with Tobacco 4 flavor e-liquid. Data from one experiment.
